# Heterogeneous Feeding Patterns of the Dengue Vector, *Aedes aegypti*, on Individual Human Hosts in Rural Thailand

**DOI:** 10.1371/journal.pntd.0003048

**Published:** 2014-08-07

**Authors:** Laura C. Harrington, Andrew Fleisher, Diego Ruiz-Moreno, Francoise Vermeylen, Chrystal V. Wa, Rebecca L. Poulson, John D. Edman, John M. Clark, James W. Jones, Sangvorn Kitthawee, Thomas W. Scott

**Affiliations:** 1 Department of Entomology, Cornell University, Ithaca, New York, United States of America; 2 Department of Entomology, University of California, Davis, Davis, California, United States of America; 3 Cornell Statistical Consulting Unit, Cornell University, Ithaca, New York, United States of America; 4 Department of Veterinary and Animal Sciences, University of Massachusetts, Amherst, Massachusetts, United States of America; 5 Department of Enteric Diseases, USAMC-AFRIMS, Bangkok, Thailand; 6 Department of Biology, Faculty of Science, Mahidol University, Bangkok, Thailand; 7 Fogarty International Center, National Institutes of Health, Bethesda, Maryland, United States of America; University of South Florida, United States of America

## Abstract

**Background:**

Mosquito biting frequency and how bites are distributed among different people can have significant epidemiologic effects. An improved understanding of mosquito vector-human interactions would refine knowledge of the entomological processes supporting pathogen transmission and could reveal targets for minimizing risk and breaking pathogen transmission cycles.

**Methodology and principal findings:**

We used human DNA blood meal profiling of the dengue virus (DENV) vector, *Aedes aegypti*, to quantify its contact with human hosts and to infer epidemiologic implications of its blood feeding behavior. We determined the number of different people bitten, biting frequency by host age, size, mosquito age, and the number of times each person was bitten. Of 3,677 engorged mosquitoes collected and 1,186 complete DNA profiles, only 420 meals matched people from the study area, indicating that *Ae. aegypti* feed on people moving transiently through communities to conduct daily business. 10–13% of engorged mosquitoes fed on more than one person. No biting rate differences were detected between high- and low-dengue transmission seasons. We estimate that 43–46% of engorged mosquitoes bit more than one person within each gonotrophic cycle. Most multiple meals were from residents of the mosquito collection house or neighbors. People ≤25 years old were bitten less often than older people. Some hosts were fed on frequently, with three hosts bitten nine times. Interaction networks for mosquitoes and humans revealed biologically significant blood feeding hotspots, including community marketplaces.

**Conclusion and significance:**

High multiple-feeding rates and feeding on community visitors are likely important features in the efficient transmission and rapid spread of DENV. These results help explain why reducing vector populations alone is difficult for dengue prevention and support the argument for additional studies of mosquito feeding behavior, which when integrated with a greater understanding of human behavior will refine estimates of risk and strategies for dengue control.

## Author Summary

Dengue, a potentially lethal infection impacting hundreds of millions of human lives annually, is caused by viruses transmitted during mosquito blood feeding. With no vaccine or treatment commercially available, understanding the underlying factors linked to virus exposure is critical for developing more effective dengue interventions. We conducted a study in an endemic region of Thailand where transmission is high and children are expected to be the non-immune, amplifying portion of the host population. We examined *Ae. aegypti* feeding patterns and risk by matching human DNA profiles in blood-fed mosquitoes to study area residents. A small number of meals matched people from the study area, suggesting that mosquitoes feed on people moving transiently through communities. People under 25 years of age were bitten less frequently than older people. We constructed network models to explore the presence of mosquito feeding “hotspots” and detected a local market “hotspot” in one study village during the high dengue transmission season. Our results provide new details on dengue vector feeding patterns and highlight the need to conduct integrated studies of vector feeding and human behavior, and virus transmission patterns in order to better understand the dengue transmission efficiency and spread.

## Introduction

Dengue is the most important arboviral diseases of humans worldwide. It occurs throughout most tropical regions. An estimated 390 million people are infected each year and approximately 96 million people suffer from clinically apparent disease annually [1], [2]. *Aedes aegypti* is the principal mosquito vector of the four dengue virus serotypes, lives in close association with humans, feeds preferentially on human blood [3]–[5] and has a tendency to ingest multiple blood meals during each gonotrophic cycle [4], [6] facilitating efficient transmission of human blood-borne pathogens. Although a tetravalent dengue vaccine is under development [7], a vaccine and anti-viral drugs are not currently commercially available. As a consequence, current dengue prevention programs are limited to control of the mosquito vector [8].

In this study, we used DNA fingerprinting to detect the individual human hosts from whom female *Ae. aegypti* took blood meals. Genetic markers have been applied to a variety of studies on mosquito feeding patterns (reviewed by Kent [9]). Coulson [10]was the first to investigate DNA fingerprinting for identifying individual human hosts. Others used a similar approach to address questions about mosquito feeding behavior and bed net efficacy [11]–[15]. In 2000, Chow-Shaffer et al. [16] used variable number tandem repeats (VNTRs) and short tandem repeats (STRs) in a pilot study to fingerprint human DNA in blood engorged *Ae. aegypti* collected in Thailand. De Benedictus et al. [14] applied the same approach to *Ae. aegypti* collected in Puerto Rico to analyze feeding patterns on 84 human hosts living in 22 houses and reported that 18% of blood meals were identified as coming from one of two people in a 36 hr time period.

Herein, we report the fine-scale details of *Ae. aegypti* blood feeding patterns on individual human hosts in a dengue endemic community. In order to better understand *Ae. aegypti*-human host interactions that underlie local DENV amplification and spread as well as human risk for infection, we used six human microsatellite markers to reconstruct blood feeding patterns of *Ae. aegypti* collected over multiple seasons, villages and years in west central Thailand. Our study objectives, were to (1) estimate the frequency at which *Ae. aegypti* bites different people in a 24 hr period; (2) determine whether human age, gender or house of residence predict the frequency at which different people are bitten and (3) evaluate the effect of advancing mosquito age on blood feeding patterns. In large outdoor field cages, we tested hypotheses concerning the impact of human host size and body position on biting behavior. In the laboratory we determined the accuracy of identifying mixed and degraded human DNA in mosquitoes that fed on more than one host. Our results indicate that frequent and heterogeneous biting by *Ae. aegypti* on residents and transient visitors and mosquito feeding/transmission hotspots are important entomologic features of dengue epidemiology.

## Methods

### Field site

Our study was conducted in four villages in northwestern Thailand: Pai Lom (16°45′N, 98°33′E) and Lao Bao (16°45′N, 98°34′E) located in Mae Pa district, 5 km north of Mae Sot in Tak Province; Mae Kasa (16°53′N, 98°37′E), located 20 km north of Mae Sot and Mae Dow (16°53′N, 98°37′E), located 20 km south of Mae Sot. Our field laboratory, a vacant village home, was located approximately 1 km from Pai Lom and Lao Bao. Descriptions of field sites, temperature and humidity during collection periods were previously described by Harrington et al. [17], [18]. Experiments were conducted during both the cool dry season (February 2000, 2001, 2002, and 2003) and warm rainy season (July 2000, 2001, and 2002). These times of the year correspond to periods of low (dry) and high (rainy) dengue transmission in Thailand [19].

### Mosquitoes

Mosquitoes were collected from inside houses using CDC backpack aspirators. Aspirator cartons were placed in plastic bags on wet ice and transported to the field laboratory where mosquitoes were anesthetized with CO_2_, chilled and sorted by species. During February 2000 and July 2000, abdomens of engorged female *Ae. aegypti* were saved for DNA analysis by smearing on filter paper, drying and placing in a sterile microcentrifuge tube. Collections from January/February 2001–January/February 2003 were preserved by homogenizing abdomens in 400 µl lysis buffer (1% SDS, 50 mM EDTA, 10 mM Tris-HCL, di H_2_O) in individual sterile microcentrifuge tubes and transported to the University of California at Davis or Cornell University for further analysis. The right wing was removed from each female and saved for body size estimation. Forceps were sterilized and air dried between each mosquito to prevent cross contamination of samples. Legs from each mosquito were removed with clean forceps and placed in a hexane washed vial for cuticular hydrocarbon (CH) age grading [20].

### DNA profiling of human population

After obtaining informed consent from study subjects, human DNA samples were collected by gently swabbing the inner cheek with a sterile wooden applicator stick. Four swabs were taken and swirled gently in lysis buffer. Each human sample was provided with a unique code indicating the person, village and date of collection. During each subsequent collection period from 2000–2003 we recorded which individuals were present, who left and who joined the study community. People from whom incomplete profiles were obtained on previous visits were re-swabbed. Participant data was numerically coded to protect the identity of subjects. Children were provided with vitamins and milk as compensation following collection of samples.

### Ethics statement

This research project was conducted with the approval of, and in accordance with, Institutional Review Boards (IRB) at the University of California at Davis (200210073), Walter Reed Army Institute of Research (752), Thai Ministry of Health Ethical Review Committee for Research in Human Subjects, and Cornell University (FWA00004513). All adult subjects provided written informed consent, and a parent or guardian of any child participant provided informed consent on their behalf.

### DNA extraction and amplification

Mosquito blood meals were extracted at UC Davis or Cornell University following methods described previously [14]. The amount of human DNA in samples was measured and distinguished from mosquito DNA using the Quantiblot Human DNA Quantitation system from Perkin Elmer (Wellesley, MA) following the manufacturer's instructions. Six human loci and a gender identification locus (AMEL)were amplified with PCR using Geneprint primers (Promega Corporation, Madison,WI). These loci were selected because they have been well characterized [21], have a high number of alleles, and relatively small PCR product lengths for greater detection probability through the mosquito blood meal digestion process. The AMEL gender identification locus (212 X, 218 Y bp) was employed, as well as the CSF1PO (291–327 bp), THO1(179–203 bp), TPOX (224–252 bp), D16S539 (264–304 bp), D7S820 (215–247 bp) and D13S317 (165–197 bp) loci. DNA was amplified in a DNA engine Dyad thermocycler (MJ Research, Waltham, MA). PCR products were run on 4–6% acrylamide-bis denaturing gels and visualized with silver staining as described previously [14].

### Determining DNA profiles

After drying, gels were examined on a light box and alleles in each mosquito blood meal were assigned a number by visual comparison to a reference 100 bp DNA ladder (Promega, Madison, WI, USA). Mosquito blood meals were included in the final analysis only if amplification was successful with at least 5 of the 6 loci. All samples with incomplete profiles after a second PCR reaction were discarded.

### Determining mosquito age

Two independent methods were used to estimate mosquito age. The first was mark–release–recapture, as described by Harrington et al. [22]. Briefly, mosquitoes were collected as pupae from natural immature development sites in the study villages. After emergence, adult females of known age were transferred to small cardboard cartons and dusted with florescent powder (DayGlo Color Corp, OH, USA). A unique color was used for each release day. Mosquitoes were released in houses within the study community after obtaining informed consent from residents of each house. Marked mosquitoes were subsequently recaptured with CDC backpack aspirators (John W. Hock Co, Gainesville, FL USA). Collected mosquitoes were transported to the field laboratory where they were anesthetized, identified to species, and examined under a dissecting microscope for florescent dust markings. Blood engorged marked and recaptured mosquitoes were processed as described above and their age was assigned based on known days since eclosion.

A second age-grading method utilized CH analysis as described by Gerade et al. [20]. Briefly, legs were removed from each specimen using clean forceps, placed in dry n-hexane washed vials, and stored until further processing at the University of Massachusetts. Hydrocarbons were extracted and analysis of legs from each mosquito was conducted against an internal standard of octadecane as described by Gerade et al. [20].

### Accuracy and limits for detecting human host DNA in mosquito blood meals

Two experiments were conducted to determine the time intervals over which DNA could be detected in a mosquito that ingested one or two (from different people) blood meals.

#### Time limits of detection of a single blood meal

Mosquito pupae were collected in water- holding containers in the villages of Lao Bao or Pai Lom during January 2001. Pupae were held in 75 mL tubes until emergence and identified to species as adults. Female and male *Ae. aegypti* were transferred to a large screened cage and held in the field laboratory under ambient conditions. Water was provided *ad libitum* for the first 2 days after eclosion. On day 3, females were allowed to feed to repletion on a human arm (one of the authors) for 15 min. Feeding time was noted and females that did not feed were discarded. Groups of 30 females were removed at 0, 6, 18, 24, 30, 36 and 42 hr after blood feeding. The experiment was replicated twice. In the field lab, abdomens of engorged mosquitoes were homogenized in lysis buffer. After extraction at Cornell University, the amount of human DNA in the blood meal was determined as described above.

#### Time limits of detection of blood from multiple human hosts in a single mosquito

In two separate multiple feeding experiments, mosquitoes were either offered two blood meals from different people with the same interval between first and second feedings (12 hr) and removed at various times following the second blood meal (experiment 1), or offered two blood meals from different people at various time intervals between first and second feedings (experiment 2) (0, 6, 12, 24 hr). One male and one female host (designated as A and B) allowed mosquitoes to bite them in this series of experiments.

In experiment 1, *Ae. aegypti* (Thai strain from the Mae Sot region, held for ca. 20 generations in the laboratory) were reared in an environmental chamber set with a variable temperature regime representing a typical Thai cool-dry season day (range 22–29°C, 8 degree days, 80% RH). Incandescent lighting was set to a crepuscular profile with 12 hr light:12 hr dark including 2 hr of simulated dawn and 2 hr of simulated dusk. Larvae were reared in trays to obtain medium body size (200/L water), fed 451 mg/larva of diet (1∶1 lactalbumin: brewer's yeast), and pupae were placed in cages for eclosion as described above. At 3–4 days of age, females were aspirated into individual glass test tubes (6 mL) and a piece of mesh was secured around the top.

Mesh-covered glass vials were carefully placed against the forearm of host A. Each mosquito was observed closely and blood feeding was interrupted by removing vials from the arm before mosquitoes could ingest a complete meal. Vials containing partially fed mosquitoes were covered lightly with water moistened paper towels and held in the environmental chamber for 12 hr until the mosquito was offered a second blood meal from host B. Each mosquito was observed directly to confirm feeding on host B. Any mosquitoes that did not feed on host B were removed from the analysis. After ingesting the second blood meal, one half of the mosquitoes were immediately frozen at −20°C and the other half were held for 24 hr before storing at −20°C. A second replicate was conducted following the same protocol except the human host order was reversed.

In experiment 2, mosquitoes were offered double blood meals from the two different people (A and B) at various time intervals between first and second feedings. Female Thai strain *Ae. aegypti* were reared as described above, placed in a 5 L cage and offered a partial meal from host A. Engorged females were divided into four subgroups of forty mosquitoes per group and allowed to feed to repletion on host B at 0, 6, 12 or 24 hr after ingesting the first blood meal. All mosquitoes were frozen at −20°C six hr after feeding on the second host (B). Mosquitoes that did not feed on both people were discarded.

Human DNA profiles were obtained with informed consent from host A and B as described above. Host DNA was extracted, amplified, and profiled as described above.

### The effect of host body mass and position on mosquito biting rates in large field cages

Large enclosures were constructed over vacant houses in Pai Lom as described previously [22]. Field cages encompassed an entire house and yard (∼10 m wide ×10 m wide ×4 m high).

Three-day-old female *Ae. aegypti* (eclosed from field collected pupae) were marked with colored dust (as described above) and released inside the field cage during July 2002. Mosquitoes were released over 4 consecutive days. A total of 353 (112, 60, 132, and 49 over days 1–4, respectively) three-day-old non-blood fed females were released inside and outside a house in the enclosure each evening. On the following day, four hosts (study authors and collaborators) entered the enclosure and remained inside for 30 min; two people were inside the house (one sitting and one lying down) and two people were outside in the same sitting or lying positions. Host location and position was rotated each day. Engorged females were collected with CDC backpack aspirators from the house and yard each day after exposure to hosts. Human DNA was extracted, amplified, profiled and matched to each participant as described above. The experiment was repeated with the same methods and 3 of the same 4 participants and one new participant during January 2003. A total of 863 (137, 75, 77, 324, 155, and 95 over days 1–6 consecutively) female *Ae. aegypti* were released into the enclosure as described above for July 2002.

### Network analysis of mosquitoes and human hosts

In order to understand whether there were spatial patterns of feeding that deviated from random (e.g. “hotspots” or “cold spots”), we used our DNA fingerprinting results to build interaction networks between mosquitoes and human hosts in the villages of Lao Bao and Pai Lom during each sampling period. Each house was considered as a node. Connections were made between nodes (houses) based on mosquito blood meals, linking the house where the mosquito was collected with the house (or houses) were human host(s) lived.

We used a traditional method to characterize networks by evaluating the derived network's topological and structural properties and comparing them with those of random networks [23]. We compared the degree distribution of each network, which was based on the number of connections that a node has; i.e., its degree. The greater the number of connections, the greater the degree. For this analysis, the majority of nodes, therefore, had approximately the same degree (close to the average k of the network). To determine if there were “hotspots” or “cold spots”, we compared the degree distribution of the observed mosquito-human biting networks, with the degree distribution obtained from 999,999 Monte Carlo randomizations (to avoid artifacts) using χ^2^ analysis at a 5% significance level.

To test the hypothesis that mosquitoes remained in or close to the houses where they were collected and people moving from house to house were bitten [18], [24], [25], we examined the spatial autocorrelation of the relationship between mosquitoes and human hosts based on observed mosquito bites from people in each house. Blood meals where the human host house of residence matched that of the mosquito collection house were defined as “resident” meals. “Non-resident meals” were designated when mosquitoes fed on someone who did not live in the mosquito collection house.

### Data analysis

Frequencies of blood meals matched to two different people were compared for experiments that examined time limits of host DNA detection. DNA profiles for mosquitoes and human cheek swabs were compared in a common data base. Questionable or partial profiles were re-amplified and re-run on gels for confirmation. All data were analyzed using two custom programs: Mosquito Matcher and/or Blood Match, which are available from the authors upon request. Both programs allowed matching analysis of data in two different excel spreadsheets. In this way the mosquito blood meal profiles could be matched to the human DNA profiles by village, season, and year. Match ID within Mosquito Matcher allowed matching of human DNA sets with each other to identify non-unique single profiles among the human population and non-unique double profiles in a theoretical mixed blood meal.

The frequency of single and multiple blood meals were initially analyzed by village of residence, season, year, and mosquito age with cross tabulations, χ^2^ test of independence and t-test. A logistic regression model was then used to model single and multiple blood feeding as a function of these variables simultaneously. Logistic regression was conducted to test the effect of village of origin, host age and sex on the probability of a person being bitten or not. A negative binomial regression model was used to test the effect of village of origin, age and sex on the number of times of a person was bitten. To analyze the effect of host age on probability of being bitten and number of times a person was bitten, age was sorted into two different types of categories. The probability of being bitten was compared among people placed in age classes by each decade from 0 years to an arbitrary upper age limit of 110. The data were also compared for people aged 0–11 representing the age group with a high probability of DENV non-immune individuals [26]. Data for the proportion of mosquitoes captured in the same house where the person they bit lived were compared across season, year and village with cross tabulations and χ^2^ tests of independence. A logistic regression model was then used to test the effect of all variables simultaneously.

In field cage experiments to compare the effect of body mass and position on host feeding patterns, a body mass parameter was calculated for each host by multiplying the height of the host (m) by their weight (kg). The body mass parameter was compared with feeding frequency across replicates. Regression analysis of the proportion of mosquitoes fed by body mass was performed.

Spatial autocorrelation for the network analysis was tested locally using G statistics [27]. For a distance d, a matrix of neighbor was generated and then local clustering was calculated using Gi(d). Due to differences on inter-house distance, the grain of the analysis was 10 m for Lao Bao and 5 m for Pai Lom; i.e., the variable d was increased every 10 and 5 m, respectively. The significance of G was evaluated using 9,999 Monte Carlo randomizations at a 5% significance level. We used this approach to determine whether popular daytime aggregation sites represent high biting risk to humans, such as homes with attached stores where residents frequented to purchase goods (local markets), were blood feeding hot spots.

All statistical analyses for comparison of blood meal frequency, season, village and year, as well as the effect of host body mass and host position, were performed in (SPSS Statistics 17.0, SPSS Inc., Chicago, IL). Statistical analyses on the interaction networks and local clustering were performed in R [28].

## Results

### Human DNA profiles

Cheek swab DNA samples were completely profiled for 676 residents from the four study villages and all study collaborators and mosquito collectors (n = 28), who periodically visited study villages. Each individual profile was unique with the exception of two identical twin boys in one village and a mother and daughter with identical profiles that lived in another village. To understand our ability to detect multiple feeding (two different people in one blood meal), we analyzed all the hypothetical combinations of two people using MatchID following the methods of DeBenedictus *et al.* [14]. A high percentage (85–94%) of these hypothetical combinations were unique (Table 1).

**Table 1:** Uniqueness of human DNA profiles obtained with informed consent from residents of 4 different villages near Mae Sot, Tak Province, Thailand.

	Village			
	Pai Lom	Lao Bao	Mae Dow	Mae Kasa
Total hosts profiled	164	240	168	232
Unique individual profiles	98%	98%	100%	100%
Unique pairs*	86%	91%	94%	90%

*Percent of unique combinations of two people, when all hypothetical combinations of two people were compared.

A total of 3,677 blood engorged mosquitoes were collected for DNA fingerprinting analysis over the course of the study. Of these specimens, we obtained complete profiles for 1,186, with the remaining samples likely too degraded to profile completely. Of the 1,186 blood meals completely profiled, 430 (36%) matched the profile of a person(s) living in a study village or a study collaborator/mosquito collector (n = 10).

### Limits of DNA detection from a single host

**Figure 1:**
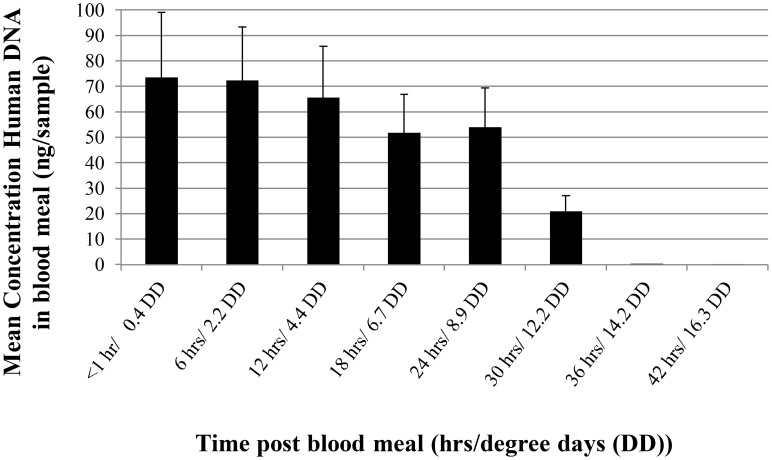
Human DNA concentration in mosquitoes over time expressed in hours and degree days (DD) for *Ae. aegypti* blood meals. DD estimates were calculated as described previously [20].

Hourly temperature during the time series experiment in January 2001 ranged from 18 to 36°C, with an average temperature of 26±0.22 SE. At time <1 hr, mosquito blood meals contained an average of 73.6±25.6 ng of human DNA. DNA concentration decreased only slightly for the next 24 hr (54 ng±15 at 24 hr/8.9 degree days (DD)) and then decreased rapidly thereafter (Fig. 1). Only trace amounts of DNA were amplified in blood meals sampled after 30 hr/11.1DD. Results for amplification success of alleles at all loci were consistent with concentration data. Complete DNA profiles from human blood were detected up to 30 hr after feeding. DNA in samples taken at 36/13.3 and 42/15.5 hr/DD was likely too degraded for amplification.

### Limits of detecting blood from two different hosts in a single mosquito

When mosquitoes took replete blood meals from two hosts in a sequence separated by 24 hr (8 DD, n = 28), only the second host DNA was detected in the blood meal, even when the order of hosts was reversed.

To simulate interrupted feeding (partial blood meals from two different hosts) mosquitoes were offered incomplete blood meals first from person A and a second blood meal from person B. With interrupted meals, partial profiles of both hosts were detected, in a small number of mosquitoes. The first host was detected in 3 of 18 (17%) mosquitoes when meals were separated by 0 hr and in 2 of 57 (4%) mosquitoes when meals were separated by 6 hr (2DD).

### Effect of host mass and position on mosquito feeding patterns in large field cages

**Figure 2:**
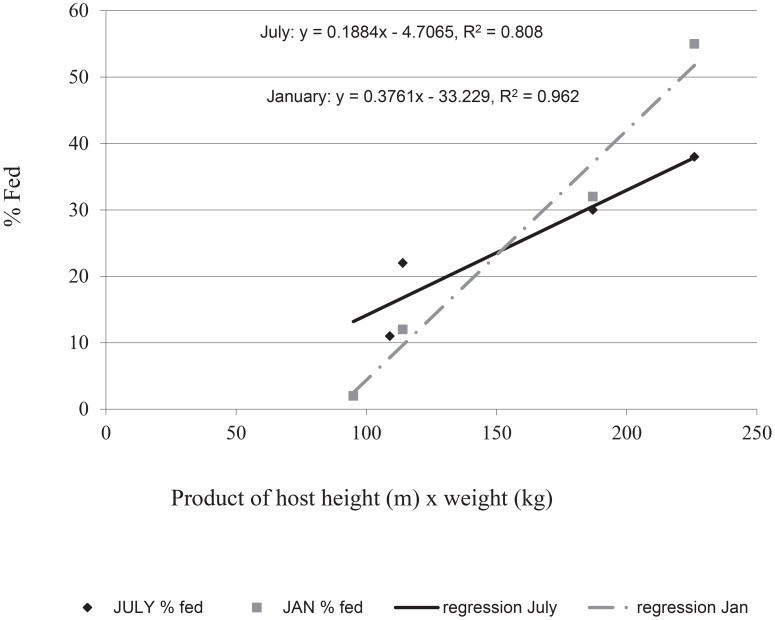
Regression of female *Ae. aegypti* feeding on hosts of known mass (product of height×weight) in large field cage studies (July and January 2003).

Feeding frequencies on four different people were directly related to host body mass in our field cage experiments. In July 2000, the majority of mosquitoes (38%) fed on the person with the largest body mass parameter (226) followed by 30% on the person with the next highest mass (187). The two smaller people (with 114 and 109 mass parameters) each were bitten 22 times (11%). In the second replicate (January 2003), 55% of all blood meals were again from the largest person, followed by 32% from the second largest person, and 12% and 2% were from people with 114 and 95 body mass, respectively. These results reveal a direct relationship between increasing host height and weight and the number of times bitten (Fig. 2) (July: adjusted R^2^ = 0.81, F = 13.6, P = 0.06; January adjusted R^2^ = 0.96, F = 75.9, P = 0.01).

### DNA fingerprinting analysis of blood meals in field-collected mosquitoes

#### Feeding by season and year

The number of collected, amplified, and matched mosquito blood meals by village, season and year are summarized in Table 2. The majority of blood meals contained single (88.6%) versus double meals (11.4%) across seasons, years and villages. We did not detect any triple blood meals. More engorged mosquitoes were collected in the rainy (78%) than dry season (11%). More total mosquitoes (fed and non-blood-fed) were collected in the rainy season. The greatest percentages of engorged mosquitoes were collected during 2001 and 2002 (Table 2).

**Table 2:** Complete and matched DNA profiles from mosquitoes collected by village, season and year in Mae Sot, Thailand (3,677 engorged mosquitoes collected; 1,186 total profiled mosquito blood meals; 430 matched to residents/collectors in the study).

		Year							Grand
		2001		2002		2003		Total	Total BM
Village	Season	Profile*	T**	Profile*	T**	Profile*	T**	Profiled*	T**
LB	Dry	2	81	0	70	5	62	7	213
	Rainy	65	116	102	237	–	–	167	353
PL	Dry	40	78	1	52	8	27	49	157
	Rainy	38	66	69	101	–	–	107	167
MD	Dry	3	53	12	60	21	25	36	138
	Rainy	21	33	13	93	–	–	34	126
MK	Rainy	20	32	–	–	–	–	20	32
Grand	Total BM	189	459	197	613	34	114	420	1186

* number profiled and matched to residents;

** total engorged mosquitoes collected and profiled; BM = blood meals analyzed.

#### Probability of being bitten

We did not detect blood in mosquitoes from 67% of the 676 village residents that were completely profiled. A relatively small portion of profiled humans were bitten by *Ae. aegypti*, ranging from 1 (n = 105, 15.5%) to 9 (n = 3, 0.4%) times (Table 3). Thirty-four people were fed on 4 or more times over the course of the study. No clear trends in age or sex of people bitten most often were detected (Table 4). The age of hosts bitten at relatively high frequencies varied from 7–96 yrs. An equal number of males (18) and females (16) were included in the high frequency group. A high proportion of these people lived in the villages of Lao Bao and Pai Lom, where our collection efforts were more frequent.

**Table 3:** Frequency of times specific hosts were bitten over the course of the study in four different villages near Mae Sot, Tak Province, Thailand, 2001–2003.

# times bitten	Number of individuals	Frequency (%)
0	453	67.0
1	105	15.5
2	59	8.7
3	24	3.6
4	14	2.1
5	9	1.3
6	7	1.0
7	2	0.3
8	0	0
9	3	0.4
Total	676	

**Table 4:** Village, age and sex of hosts bitten more than 4 times.

Frequency bitten (# times)	Village	Sex	Age
4	Lao Bao	F	7
4	Pai Lom	F	8
4	Pai Lom	F	25
4	Pai Lom	F	29
4	Mae Dow	F	32
4	Pai Lom	F	34
4	Lao Bao	F	37
4	Pai Lom	F	40
4	Lao Bao	M	18
4	Pai Lom	M	31
4	Pai Lom	M	31
4	Lao Bao	M	34
4	Lao Bao	M	43
4	Pai Lom	M	44
5	Lao Bao	F	11
5	Lao Bao	F	18
5	Pai Lom	F	31
5	Mae Dow	F	35
5	Lao Bao	F	42
5	Lao Bao	M	17
5	Mae Dow	M	29
5	Pai Lom	M	34
5	Lao Bao	M	45
6	Lao Bao	F	15
6	Pai Lom	F	80
6	Pai Lom	M	32
6	Lao Bao	M	59
6	Mae Dow	M	64
6	Pai Lom	M	77
6	Mae Dow	M	96
7	Lao Bao	M	62
7	Lao Bao	M	67
9	Lao Bao	F	37
9	Pai Lom	M	11
9	Lao Bao	M	38

The probability of being bitten varied significantly by village (Logistic regression, Wald = 47.2, df = 4, P<0.001). Feeding rates were significantly higher for profiled people in the villages of Pai Lom and Lao Bao (χ^2^ = 47.5, P<0.001), where the majority of profiled mosquitoes were collected and 46.3% and 41.0% of the village residents were bitten, respectively. No significant difference was detected for the probability of being bitten for females (34.6% of all female hosts) versus males (31.7% of all male hosts) (χ^2^ = 0.65, P = 0.42). Of the total engorged mosquitoes profiled, 48% (566) were from Lao Bao, 27% (324) from Pai Lom, 22% (264) from Mae Dow, and 3% (32) from Mae Kasa (Table 2).

#### Multiple blood meals

**Figure 3:**
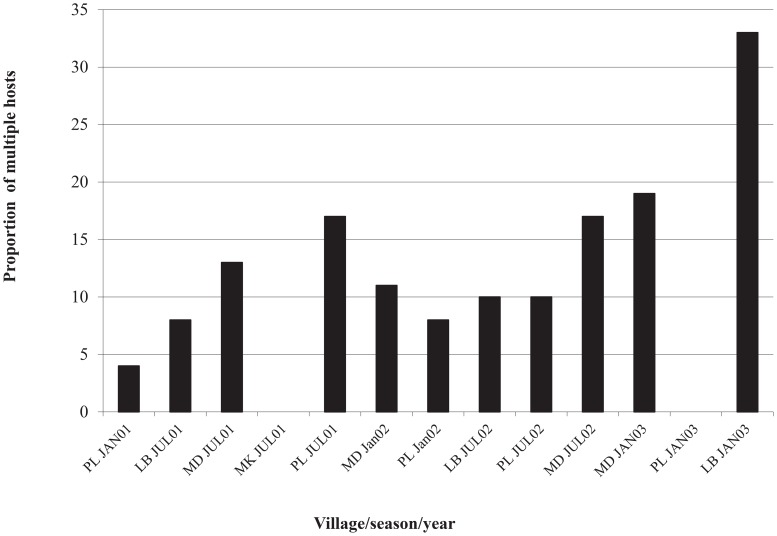
Proportion of blood from multiple hosts detected in 430 amplified and matched blood meals over multiple villages, seasons and years in Mae Sot, Thailand. Villages designations are: PL = Pailom, LB = Lao Bao, MD = Mae Dow.

Multiple feeding rates (i.e., feeding on 2 different people in a 30 hr time period) ranged from 0–32% depending on the season, village and year (Fig. 3). The average multiple feeding rate detected was 10–13%. Slightly more double blood meals were collected in 2003 (20%) compared with 2000–2002 (χ^2^ = 7.09, df = 3, P = 0.07). No significant differences were detected among double blood meals taken during the rainy (88.4% single, 11.6% double) versus dry season (89.2% single, 10.8% double; χ^2^ = 0.05, df = 1, P = 0.50) or by collection village (χ^2^ = 4.99, df = 3, P = 0.17) when controlled for total mosquitoes collected. These results are inconsistent with the hypothesis that dengue transmission peaks during the rainy season due to increased multiple feeding rates by *Ae. aegypti* [29]. Our results, however, probably underestimate the rate of multiple feeding because the period of efficient detection of DNA from two different hosts in our study was short even when two meals were separated by 6 hr or less.

We estimated the daily feeding rate on different people using previously described methods [5]. The average percent of freshly blood-engorged females (indicating they had taken a blood meal on the day of capture) over the study period was 33%. By combining our estimates of multiple feeding on different people within 24 hr (10–13%) engorgement rate (33%) and assuming that meals on different days came from different people, we estimate that 43–46% of females bit a different person each day.

Seventy-two percent and 81% of multiple blood meals from the dry and rainy season, respectively, were from people who lived in the same house. The remainder where from people living in adjacent houses, with the exception of nine blood meals. For those nine, eight contained DNA from people that lived in another village (all were from the adjacent villages, either Lao Bao or Pai Lom, which were separated by approximately 300 m) and one contained blood from one of our mosquito collectors. Although we did not monitor human movement within and between the villages, we observed movement and house visitation between residents of the two adjacent villages of Lao Bao and Pai Lom.

#### Feeding by host age

We examined the frequency at which different aged people were bitten in two different ways. First, host age was compared at 10 yr intervals (Table 5). Fewer people in younger age categories were fed on than expected. Second, we examined feeding rates on children up to 13 yrs (which represents an age class that traditionally has high rates of DENV infection [26]). In general, people 25 years old and younger were fed on less often than those older than 25 (χ^2^ = 11.0, df = 3, P = 0.012). A negative binomial regression of the number of bites by age, sex and village, indicated significant differences by age (LLR χ^2^ = 28.8; d.f. = 1; p<0.001) and village (LLR χ^2^ = 83.2;d.f. = 4; p<0.001).

**Table 5:** Percentage of blood meals from people by decadal age class for all blood meals.

Age category	% not bitten (n)	% bitten (n)*	Total
0–9	75.6% (93)	24.4% (30)	123
10–19	65.7% (65)	34.3% (34)*	99
20–29	76.9% (70)	23.1% (21)	91
30–39	60.0% (81)	40.0% (54)*	135
40–49	65.4% (68)	34.6% (36)*	104
50–59	76.9% (40)	23.1% (12)	52
60–69	43.2% (19)	56.8% (25)*	44
70–79	53.3% (8)	46.7% (7)*	15
80–110	62.5% (5)	37.5% (3)	8
**Total**	**449**	**222**	**671**

* feeding patterns are significantly different from random (χ^2^ = 26.28, df = 8, P = 0.01).

#### Feeding patterns by mosquito age

*Mark-release-recapture*: Human DNA fingerprints were obtained for 20 marked mosquitoes of known age that were released and recaptured in the study area. Eighteen of the mosquitoes aged 4–12 days took a single blood meal and three (aged 5–6 days) had ingested blood from more than one person (Table 6). A sample size of 3 mosquitoes that fed more than once was too small to detect vector age-specific feeding patterns. All but 3 mosquitoes fed on people that lived in the village where they were collected. The 3 exceptions fed on someone living in another village or on one of our study collectors.

**Table 6:** Feeding patterns by vector age determined from mark-release-recapture experiments in Thailand, 2001.

Season	Mosquito Release Village	Mosquito Age at Capture (days)	Host 1 Sex	Host 1 Age	Host 1 House*(S/D)	Host 2 Sex	Host 2 Age	Host 2 House*(S/D)
July/Rainy	Lao Bao	4	F	47	S			
July/Rainy	Lao Bao	4	M	45	S			
July/Rainy	Lao Bao	6	F	59	S	M	4	D
July/Rainy	Lao Bao	6	F	59	S			
July/Rainy	Lao Bao	7	M	48	D			
July/Rainy	Lao Bao	9	M	48	S			
July/Rainy	Lao Bao	9	F	35	S			
July/Rainy	Lao Bao	11	F	11	S			
July/Rainy	Lao Bao	5	F	12	S			
July/Rainy	Lao Bao	5	M	30	D	F	12	S
July/Rainy	Lao Bao	6	F	12	S	M	40	S
July/Rainy	Lao Bao	9	M	14	S			
July/Rainy	Mae Kasa	7	M	51	S			
July/Rainy	Mae Kasa	9	M	30	D			
July/Rainy	Mae Kasa	11	M	18	S			
July/Rainy	Mae Kasa	4	F	10	S			
July/Rainy	Mae Kasa	6	F	10	S			
Jan/Dry	Pai Lom	8	M	25	S			
Jan/Dry	Pai Lom	10	M	34	D			
Jan/Dry	Pai Lom	11	M	66	S			

*Host house same as mosquito release house = S; different from mosquito release house = D.

### Cuticular hydrocarbon methods

A total of 47 females with complete DNA profiles were age-graded using cuticular hydrocarbon ratios. A total of 43 females ingested a single blood meal and 4 took double blood meals. No significant difference in age by blood meal number was detected. The mean age for females ingesting blood from 1 person was 5.4 days (±0.35, range <1–12). The mean age for females that fed on more than one person was 5.4 days (±0.45, range <1–13).

### House resident vs. non-resident blood meals

The number of “house resident” and “non- resident” meals per season and village is presented in Table 7. Overall, the majority of mosquitoes were captured in the same house where the person they bit lived (64.6%). More mosquitoes were captured in the same house as where their blood hosts lived during the rainy (69.7%) than dry season (45.1%, χ^2^ = 19.2, df = 1, P<0.0001). Significant differences also were detected by year and village. The greatest percentage of mosquitoes were captured in the same house where their hosts lived during 2001 and 2002 (72.7% and 65.6%, respectively, χ^2^ = 25.7, df = 3, P<0.0001). Greater than 62% of mosquitoes were captured in the same house as the person they bit in the villages of Mae Kasa, Lao Bao, and Pai Lom than Mae Dow, where the smallest number of profiled mosquitoes were collected (49.3%) (χ^2^ = 19.7, df = 3, P<0.0001). Logistic regression analysis revealed a significant effect of year (Wald = 0.000, df = 1, P<0.0001) and village (Wald = 0.00, df = 1, P<0.0001).

**Table 7:** Number of mosquito blood meals taken from a house resident and non-resident and number of houses with blood meals that were identified to a person per season per village.

Village	Season	House resident meals	Non-resident meals	Houses with meals detected	Houses with no meal detected
Pai Lom	Dry 2001	12	4	10	28
Mae Dow	Dry 2002	3	0	2	19
Lao Bao	Dry 2003	0	6	2	53
Pai Lom	Dry 2003	4	1	5	33
Mae Dow	Dry 2003	3	0	2	19
Lao Bao	Rainy 2001	48	6	21	34
Pai Lom	Rainy 2001	13	5	12	26
Mae Kasa	Rainy 2001	18	0	9	59
Mae Dow	Rainy 2001	7	1	3	18
Lao Bao	Rainy 2002	134	56	31	24
Pai Lom	Rainy 2002	46	10	23	15
Mae Dow	Rainy 2002	7	0	5	16

### Spatial analysis and clustering

**Figure 4:**
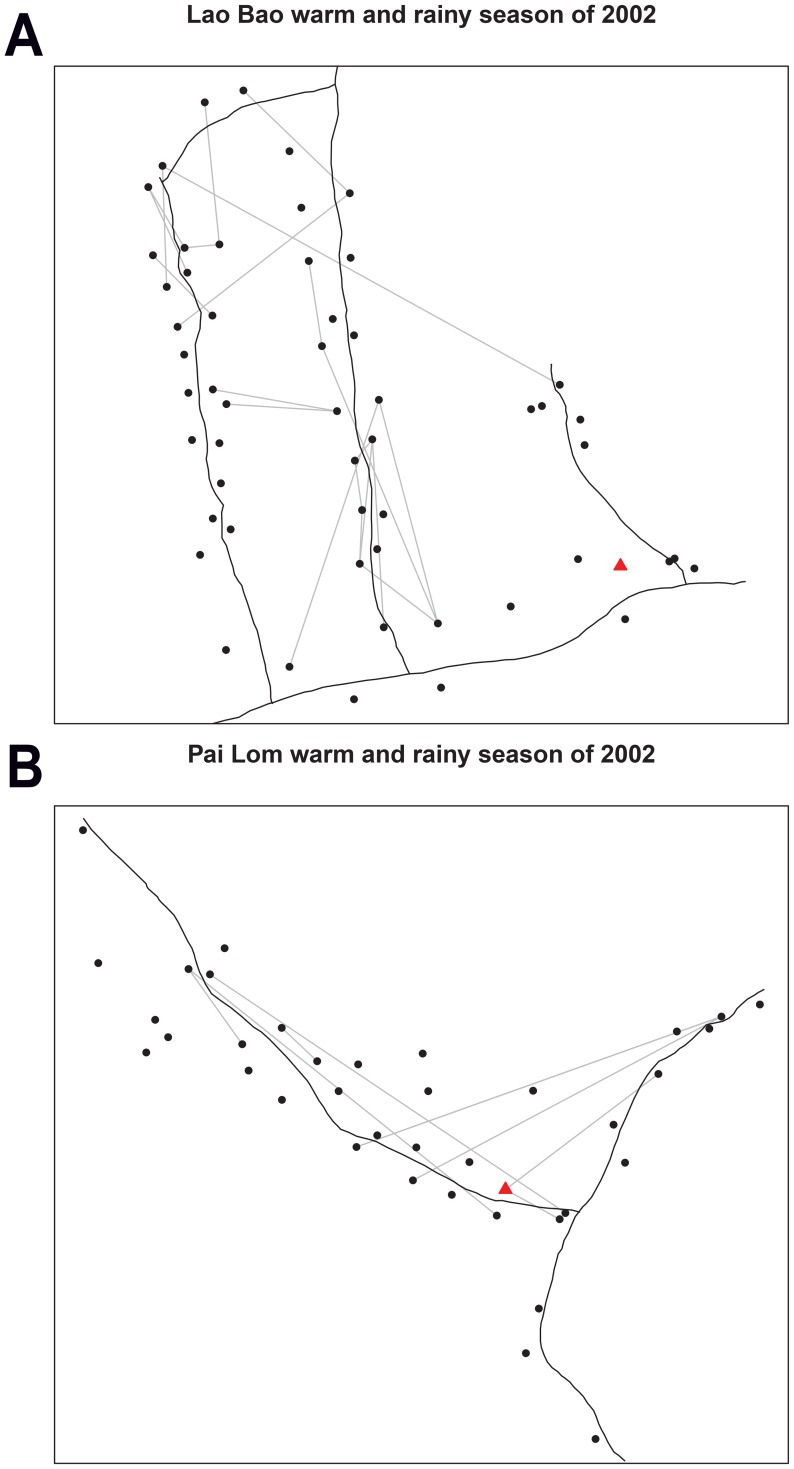
Interaction networks for Pai Lom (bottom) and Lao Bao (top) during the rainy season of 2002. Black lines represent roads. Dots represent the location of households, triangles indicate local markets and gray lines represent mosquito meals from one house connected to human hosts in another house.

Observed biting networks are presented in Fig. 4. Analysis of the degree distribution (Table 8) revealed no significant clustering of feeding patterns for interaction networks in Pai Lom over all sampling events. Significant house-level clustering patterns were observed, however, for Lao Bao for the 2002 rainy high dengue transmission season and the 2003 dry low dengue transmission season. In both cases more households were disconnected with more localized feeding patterns on house residents and less connections to feeding on hosts in other houses.

**Table 8:** Observed mosquito-human interaction networks versus randomizations.

Village	Season	Year		
		2001	2002	2003
Lao Bao	Dry	—	—	+0; +5
				−1
	Rainy	Random	+0; +6; +12; +14	—
			−1; −3	
Pai Lom	Dry	Random	—	Random
	Rainy	Random	Random	—
Mae Dow	Dry	—	+0	+0
			−1	−1
	Rainy	Random	+0	—
			−1	
Mae Kasa	Dry	—	—	—
	Rainy	+0	—	—
		−1		

Results from the comparison of observed mosquito-human interaction networks against 999,999 randomizations, with a 5% significance level. “—” = no data. Random means there was no significant difference between the observed data and the randomizations. + (−) signs indicate that the observed network has more (less) nodes with the degree indicated by the number next to the sign. Hence, the network corresponding to the dry 2003 season in Lao Bao, displayed more isolated nodes (+0), more nodes with 5 connections, and less nodes with 1 connection than the randomizations.

**Figure 5:**
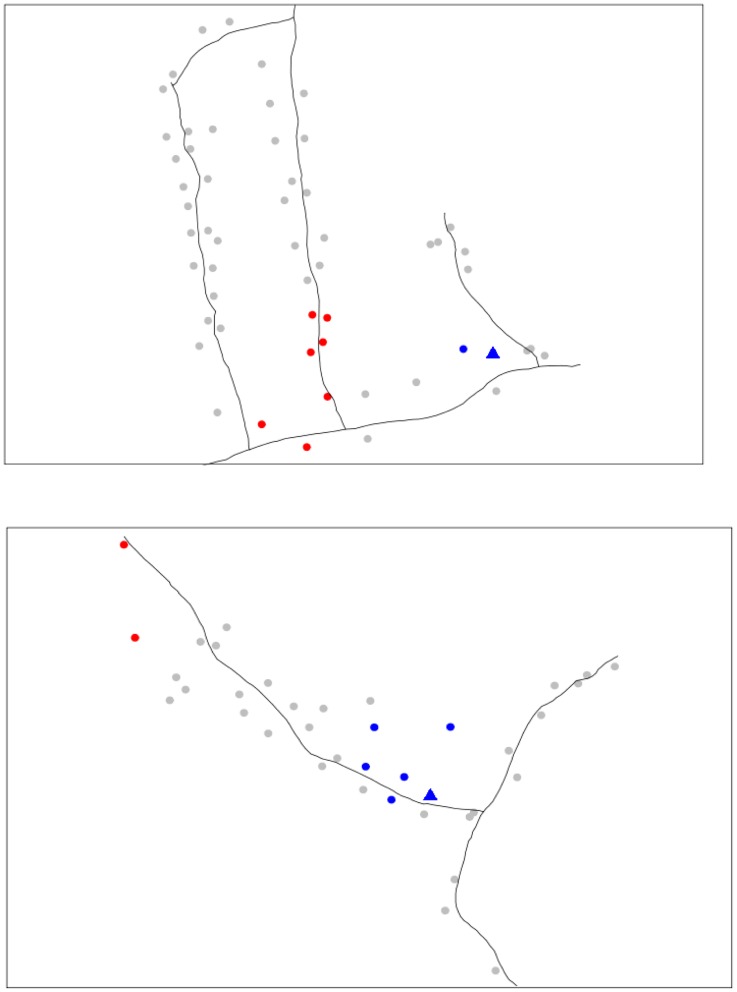
Local clustering for house resident meals for Lao Bao (top) and Pai Lom (bottom) during the rainy season of 2002 with neighbors defined at 40 m and significance evaluated using 9,999 randomizations. Red and blue indicate the presence of hot and cold spots, respectively. Triangle indicates the local market. Black lines represent roads.

Clustering patterns for mosquito biting was associated with the presence of hot and cold spots in the villages (Fig. 5). These demonstrated more (hot) and less (cold) than expected resident and non-resident blood meals, respectively. Hot and cold spots were detected at several scales during most of the sampling events in both Lao Bao and Pai Lom (Table S1.A–D). Values in Table 8 indicated with parenthesis and a star symbol were those that included the village local market. Values report the interval of distances (m) where significant clustering of bites was detected. For example, in Table 8 B for Lao Bao during the 2001 warm rainy season, hot spots were detected with neighboring houses if the distance among them was 30–60 m, 80 m, and 100–130 m. For 60 m, the clustering included the local market. Although statistically significant, reported clusters at scales bigger than 60 for Lao Bao may not be accurate because of statistical constraints due to the size of the village which was smaller than the two larger cluster sizes. For Lao Bao, local clustering for “resident” bites was observed only during the rainy seasons (2001 and 2002), and in both cases hot spots included the local market. Local clustering for “non-resident” bites was detected for all the sampling events, and the local market was included when neighborhoods were calculated at 80 m or less.

For the cool dry low dengue transmission season of 2001 in Pai Lom, the local market was included in the households detected as cold spots for both the “resident” bites (at scales 95–120 m), and the “non-resident” bites (at 65–125 and 160–185 m). In addition, spatial linear regression analysis confirmed a global trend of increasing mosquito biting activity northeast of the local market (p = 0.005); i.e., increasing latitude (p = 0.03), decreasing longitude (p = 0.02).

## Discussion

*Ae. aegypti* feeds preferentially on humans, and is adapted to living in close proximity to humans, often resting and blood feeding within human dwellings [3], [29]–[31]. This mosquito vector is permissive to infection and transmission of DEN viruses, although vector competence varies with viral, environmental and mosquito genetic factors [32], [33]. The burden of dengue in countries such as Thailand where we conducted our study, is high [26]. Despite this high force of transmission, *Ae. aegypti* is often found in surprisingly low densities in and around human households and mosquito DENV infection rates during epidemics are typically low (3–7% in Singapore; <1.0% in Khamphaeng Phet,Thailand; 4.0% in Southern India [26], [34], [35], but can vary on fine spatiotemporal scales [36].

This entomological paradox raises important questions about mosquito feeding behavior and the factors that drive DENV transmission. The goal of our study was to investigate whether heterogeneities in *Ae. aegypti* blood feeding behavior are consistent with epidemiologically meaningful patterns, evidenced by seasonal shifts in multiple feeding patterns, variation by host age, sex, and location of blood feeding hotspots within communities. To do this we evaluated mosquito human feeding patterns over a long time period that included multiple seasons and years in a dengue endemic setting.

Previous studies, conducted on a smaller scale, reported heterogeneities in human feeding patterns for *Ae. aegypti* [6], [14], [16], *Culex quinquefasciatus* [13], *Anopheles gambiae*, and *An. funestus* [12], [37] with some results similar to ours, such as frequent feeding patterns on individual hosts [14], [38] and a relationship between host body size and biting frequency [37]. The majority of blood meals in our study were from single hosts that were only detected in mosquitoes once or twice, but some hosts were fed on frequently. For example, three people were bitten nine times over the course of our study. No clear patterns emerge regarding why certain people in our study were fed on so frequently, even though we examined a diversity of ages and body sizes. These heterogeneities in biting trends suggest a role for chemical or environmental cues in human host attraction [39] and/or differences in the opportunities for mosquitoes to encounter and bite different people. Host blood detected multiple times in mosquitoes may represent individuals with the potential to contribute more to transmission than others. The relative impact of these individuals as potential “super spreaders” on the dynamics of dengue outbreaks merits additional study [40].

Based on laboratory optimization experiments, our ability to detect more than one host in a single blood meal was limited if the time interval between the two host blood meals was greater than 6 hrs. Additional experiments revealed that the likelihood of identifying a multiple blood meal decreases when mosquito feeding was interrupted. Together, these results suggest that we may significantly underestimate multiple blood feeding within a gonotrophic cycle (typically 3–6 days), and when meals are interrupted due to host defenses and other factors. Given these limits of human DNA detection in a mosquito blood meal our field estimates for multiple feeding likely represent minimum frequencies for *Ae. aegypti*.

Although we completely profiled human DNA from a large number of mosquitoes (n = 1,186), 64% did not match anyone in the study community. Even in our most isolated village, Pai Lom, and taking into account error rates (approximately 0.20–0.26), we were not able to match up to 28% of human hosts in blood meals collected. This result likely was not due to *Ae. aegypti* feeding on non-human blood meals. Numerous studies have shown low non-human blood feeding rates for *Ae. aegypti* in Thailand and elsewhere [6], [31], [41], [42], including the villages studied here. In addition, the majority of the samples analyzed with our slot blot procedure contained human DNA. Although our house resident vs. non-resident analysis revealed that most mosquitoes fed on house residents, our comparison was only for those non-residents living in the same village that could be matched to blood meals.

Our laboratory studies generated incomplete profiles as a result of increasing blood meal digestion time and with multiple meals. Direct sequencing of mosquito blood meals in future studies will likely improve the quality and accuracy of profiled blood meal data.

Although we did not monitor human movement in our study we suspect that a considerable number of people were moving transiently through the study communities, either to conduct daily business, work during peak harvest times, or visit friends and relatives. Given our knowledge of the limited short-range movement of *Ae. aegypti* [43], and recent work on human movement patterns and DENV transmission by Stoddard et al. [24], [25], it is reasonable to speculate that a significant proportion of mosquitoes fed on visitors entering village houses in our study area. *Aedes aegypti* biting human visitors is a mechanism by which virus could be introduced into and/or carried away from the communities we studied. If feeding rates are high in an introduction zone, dengue transmission “hot spots” could occur, contributing to the focal nature of dengue cases [36], [44]. In our study a local market in one village represented a hot spot during two high dengue transmission seasons. Feeding on visitors may explain how DENV is introduced into communities, while localized feeding hotspots and network biting patterns may explain the focal nature of DENV outbreaks [36], [44]. In addition, more work is needed to understand the factors that influence of mosquito biting clusters. For example, those factors may include the number of people living in a house, the type, quantity, and microclimate of optimal mosquito resting sites and other refugia, as well as landscape barriers to mosquito movement.

Due to *Ae. aegypti*'s propensity to bite older people, in regions where DENV transmission is low, unstable or a novel virus serotype or genotype is introduced, a significant portion of susceptible older individuals may be at higher risk of infection than younger people. In areas of hyperendemic transmission, such as our study area in Thailand, children and young adults are at greater risk of infection because older people will have already been infected with all four virus serotypes [26]. We found a significantly lower feeding frequency on people under 25 yrs of age (approximately 10% of blood meals) than expected if bites were random. Our field cage studies reinforced this result, demonstrating a strong feeding preference of *Ae. aegypti* for larger hosts when given a choice, consistent with other studies [37], [45]. A range of choices may not be available, however, at all times for host seeking *Ae. aegypti*. For example, transmission may occur at school where the majority of hosts are small. Or, if a child is the first to return home after a day at school, he/she may be the only person available to bite. We did not collect mosquitoes from schools, and our methods may not have been sensitive enough to detect fine scale timing of mosquito feeding that would convey opportunistic feeding on naïve hosts such as the scenario of a child coming home from school before adults. House scale differences are likely important in DENV transmission, especially when considering naturally low mosquito infection rates (approximately, 1% in or around the home of an infected person and 0.1% across communities [36], [46]). These results may help explain why DENV outbreaks can rapidly expand in areas of virus introduction and low transmission stability if mosquitoes feed preferentially on susceptible adults.

One way to overcome relatively small probabilities of mosquito infection and transmission is with high multiple feeding rates [30], especially by infectious mosquitoes. We estimate from our detection of multiple blood feeding on different human hosts, that nearly half of the engorged mosquitoes we collected (43–46%) fed more than once in an egg-laying cycle, which can last from 3–5 days in the study area depending on the season (Harrington, unpublished data). This frequent biting rate coupled with high survival may overcome low DENV mosquito infection rates and relatively low feeding frequency on epidemiologically naïve hosts. Although we did not detect an age related trend in frequency of feeding, our sample size of feeding rates in older mosquitoes was too small to be conclusive. Future studies with the sole focus of understanding age-related host biting patterns would help clarify this enigma.

Our results do not support some explanations for fluctuations in DENV transmission, such as higher feeding rates during the high dengue transmission season [29], [47]. However, given the variation we observed, our data may not have had the precision to detect these differences. Similarly, we did not detect higher rates of feeding on naïve (and, therefore, potentially susceptible or infectious) hosts [47]–[49]. Interestingly, we did observe higher rates of in-house resident biting by mosquitoes during the rainy season, which correlated with the high dengue transmission season in this region of Thailand.

We did not investigate mosquito biting by time of day or the amount of time people spent in their homes where they would be at risk of being bitten by resident mosquitoes. Future studies can focus on feeding patterns in the various places outside of home that people visited (including schools, homes of friends and relatives, and gathering places) and how the risk of these aspects of dengue vector biology may reveal epidemiologically important trends.

Our results highlight the importance of identifying local hotspots for mosquito biting. If hotspots can be identified, focal insecticide spraying could be more effective and cost less for reducing DENV transmission than treating entire villages. More emphasis should be placed on strategies that identify and test the epidemiologic significance blood feeding hot spots.

Our results help explain why vector control alone is difficult for dengue prevention, due to the very low mosquito population levels that may need to be achieved with heterogeneous biting mosquitoes. *Aedes aegypti* is a highly efficient virus vector because of its frequent and non-random interactions with human hosts. Consequently, relatively few *Ae. aegypti* females can lead to unacceptable levels of DENV transmission. Additional studies of mosquito feeding behavior, which when integrated with a greater understanding of human behavior, are needed to refine estimates of dengue risk and to improve strategies for its control.

## Supporting Information

Table S1 A – Intervals of significant distances where significant clustering (hotspots) was detected for blood meals from house residents; S1.B - Intervals of significant distances where significant clustering (cold spots) was detected for blood meals from house residents; S1.C – Intervals of significant distances where significant clustering (hot spots) was detected for blood meals from house residents; S1.D - Intervals of significant distances where significant clustering (cold spots) was detected for blood meals from house residents.
(DOCX)
