# Supplementary material for: Heterogeneous Feeding Patterns of the Dengue Vector, Aedes aegypti, on Individual Human Hosts in Rural Thailand
Source: PLoS Negl Trop Dis. 2014 Aug 7;8(8):e3048. doi: 10.1371/journal.pntd.0003048 (PMC4125296; doi:10.1371/journal.pntd.0003048)
Supplement: Table S1 — A – Intervals of significant distances where significant clustering (hotspots) was detected for blood meals from house residents; S1.B - Intervals of significant distances where significant clustering (cold spots) was detected for blood meals from house residents; S1.C – Intervals of significant distances where significant clustering (hot spots) was detected for blood meals from house residents; S1.D - Intervals of significant distances where significant clustering (cold spots) was detected for blood meals from house residents. (DOCX) [file pntd.0003048.s001.docx]

Table S1.A – Intervals of significant distances where significant clustering (hotspots) was detected for blood meals from house residents

| Village | Season | Year | | |
| --- | --- | --- | --- | --- |
|  |  | 2001 | 2002 | 2003 |
| Lao Bao | Cold Dry | --- | --- | No clustering detected |
|  | Warm Rainy | 30-60 & 80 & 100-130  (60)* | 30-190  (40-50)* | --- |
| Pai Lom | Cold Dry | 45-170 | --- | 45-65 & 235-265 |
|  | Warm Rainy | No clustering detected | 45-70 | --- |

Table S1.B - Intervals of significant distances where significant clustering (cold spots) was detected for blood meals from house residents

| Village | Season | Year | | |
| --- | --- | --- | --- | --- |
|  |  | 2001 | 2002 | 2003 |
| Lao Bao | Cold Dry | --- | --- | No clustering detected |
|  | Warm Rainy | 40-130 | 40-70 & 100 & 120-190 | --- |
| Pai Lom | Cold Dry | 45-205  (95-100)* | --- | 55-220 |
|  | Warm Rainy | 140 & 165-170 & 205 | No clustering detected | --- |

Table S1.C – Intervals of significant distances where significant clustering (hot spots) was detected for blood meals from house residents

| Village | Season | Year | | |
| --- | --- | --- | --- | --- |
|  |  | 2001 | 2002 | 2003 |
| Lao Bao | Cold Dry | --- | --- | 30-110 & 130  (110)* |
|  | Warm Rainy | 30-100 | 30-120 | --- |
| Pai Lom | Cold Dry | 45-85 | --- | 45-65 & 75-90 |
|  | Warm Rainy | 160 | No clustering detected | --- |

Table S1.D - Intervals of significant distances where significant clustering (cold spots) was detected for blood meals from house residents

| Village | Season | Year | | |
| --- | --- | --- | --- | --- |
|  |  | 2001 | 2002 | 2003 |
| Lao Bao | Cold Dry | --- | --- | 30-110 |
|  | Warm Rainy | 30-110 & 130-140 | 40-50 & 70-130 | --- |
| Pai Lom | Cold Dry | 60-265  (65-125 & 160-185)* | --- | 145 |
|  | Warm Rainy | 150 | No clustering detected | --- |

Cells with “---” indicate no data. Values indicated with ( )* include the local market.
